# Sensitivity and specificity of point-of-care circulating Cathodic antigen test before and after praziquantel treatment in diagnosing *Schistosoma mansoni* infection in adult population co-infected with human immunodeficiency virus-1, North-Western Tanzania

**DOI:** 10.1186/s13690-018-0274-4

**Published:** 2018-06-25

**Authors:** Humphrey D. Mazigo, Stella Kepha, Safari M. Kinung’hi

**Affiliations:** 1 Department of Medical Parasitology and Entomology, School of Medicine, Catholic University of Health and Allied Sciences, P.O. Box 1464, Mwanza, Tanzania; 2 London School of Hygiene and Tropical Medicine, London, UK; 3 National Institute for Medical Research, Mwanza Research Centre, P.O. Box 1462, Mwanza, Tanzania

**Keywords:** *Schistosoma mansoni*, HIV-1, Point-of-care circulating Cathodic antigen, Sensitivity, Specificity, Adult, Tanzania

## Abstract

**Background:**

The effect of Human Immunodeficiency Virus-1 (HIV-1) on CD4^+^ Th_2_ cells is hypothesized to affect parasitological diagnosis of *Schistosoma mansoni* using Kato Katz technique. Thus, the use of more sensitive technique such as Point-of-Care Circulating Cathodic Antigen (POC-CCA) test is recommended. However, the sensitivity of this diagnostic test in diagnosing *S.mansoni* infection and the usefulness of it in monitoring efficacy of praziquantel drug in presence of HIV-1 co-infection remains inconclusive. The Primary objective of the present study was to assess accuracy of the POC-CCA test in diagnosing *S.mansoni* infection before and after praziquantel treatment in adult population co-infected with HIV -1.

**Methods:**

A prospective longitudinal study was conducted among individuals aged 15–55 years at Igalagala village, north-western Tanzania. At baseline and 4 weeks after treatment, a single stool and urine samples were collected from each participants. Kato Katz (KK) technique and Point-of-Care Circulating Cathodic Antigen tests were used for diagnosis of *Schistosoma mansoni.*

**Results:**

At baseline, based on KK and POC-CCA, the prevalence of *S.mansoni* was 57.8% (95%CI: 52.9–62.4) and 87.5% (95%CI: 83.9–90.4). Based on KK technique and POC-CCA test, 3.6% and 5.7% of the study participants were co-infected with *S.mansoni* and HIV-1. At baseline, in the general population, the sensitivities of POC-CCA test using KK technique and combine gold standard were 96.3%(95%CI: 93.1–98.3) and 97.6%(95%CI:95.5–98.9) respectively. In the HIV-1 seropositive group, at baseline, the sensitivities of POC-CCA test using KK technique and combined gold standards, were 93.3%(95%CI:68.1–99.8) and 96%(95CI%:79.6–99.9). Four weeks after treatment, in general population, the sensitivities of POC-CCA test using KK technique and combined gold standards were 47.8%(95%CI:26.8–69.4) and 84.4%(95%CI:74.4–91.7). In the HIV-1 seropositive group, using KK technique, the sensitivity was 100% (95%CI:2.5–100).

**Conclusion:**

The sensitivity of POC-CCA in diagnosing *S.mansoni* infection was higher than KK technique in adult individuals likely to have low infection intensity and co-infected with HIV-1. However, its sensitivity decreases following praziquantel treatment but remained higher than Kato Katz technique. If the goal of the post-treatment is to identify uncured individuals, then POC-CCA test offers the best choice.

## Background

In the past three decades, epidemiological studies have demonstrated an overlap of Human Immunodeficiency (HIV-1) and *Schistosoma mansoni* infections in Sub-Saharan Africa [1, 2], leading to co-infection in proportion of individuals living in highly endemic regions [3, 4]. It is hypothesized that helminths infections such as *Schistosoma mansoni* may exacerbate HIV-1 transmission or progression to AIDS in Africa [2, 3]. This is based on observations that infection with these parasites cause a chronic stimulation of the immune system with a strong bias towards a type 2 CD4^+^helper T cell (Th_2_) response [3, 5]. This in turn affects the ability of the host’s immune system to control HIV-1 replication [3]. Conversely, HIV-induced destruction of CD4^+^ T cells, especially those that secrete type 2 cytokines [6, 7]; this may affect granuloma formation and alter egg excretion efficiency.

Studies in suppressed animal models has demonstrated that the excretion of *S.mansoni* eggs is immune dependent, and that T-cells, specifically anti-eggs Th_2_ response [8] are necessary for the transposition and excretion of eggs from the host blood stream into the intestinal lumen [7]. HIV-1 infection has been demonstrated to affect *S.mansoni* eggs excretion efficiencies [9]. This observation have been described to impact on diagnosis of *S.mansoni* infection using Kato Katz technique, which depend on examination of the parasite eggs [9]. This may lead to underestimation of the prevalence and intensity of infection before and after praziquantel treatment in adult population co-infected or not with HIV-1 [9]. The potential for HIV-1 to affect *S. mansoni* eggs excretion makes the detection of circulating schistosome antigens released insitu particularly important in detecting *S. mansoni* infection [10]. In this context, a Point-of-Care Circulating Cathodic Antigen test has been developed as an alternative test to the Kato Katz technique. This test has been extensively assessed in sub-Saharan Africa [10, 11]. However, the results on the sensitivity of circulating antigen detection tests in adult individuals co-infected or not with HIV-1 evaluated before and after praziquantel treatment have yielded contrasting results [12, 13]. The discrepancies observed between these studies calls for further studies to further evaluate the sensitivity of POC-CCA test in the adult population and assess its effectiveness in evaluating mass drug administration treatment programs in adult population. In this context, the primary objective of this study was to assess the sensitivity and specificity of Point-of-Care Circulating Cathodic Antigens (POC-CCA) test in diagnosing *S. mansoni* infection in comparison to parasitological Kato Katz technique (regarded as “gold standard”) and a combined gold standard (infection-positive by either Kato Katz technique or POC-CCA-positivity (assuming 100% specificity of the POC-CCA test) [14] among adult population living in a fishing village of north-western Tanzania, either co-infected or not with HIV-1. Secondarily, the study assessed the usefulness of Point-of-Care Circulating Cathodic Antigen test in monitoring the efficacy (CCA clearance at 4 weeks following treatment) of praziquantel drug in presence of HIV-1 infection among adult population.

## Methods

### Study area

The study area is described in details in [15, 16], briefly, the present study was conducted at Ilemela district, Mwanza region, located at 32-34°E and 2-4^0^S, on the southern shores of the Lake Victoria, north-western Tanzania. Specifically, the study was conducted at Igalagala village located at shoreline of the Lake Victoria. The choice of this village was based on its close proximity to the lake, high prevalence and intensity of *S. mansoni* infection [16] and high HIV-1 prevalence [15]. The majority of the populations are of Sukuma tribe and other are migrating tribes such as Wakerewe, Wajita and Wakara. The main economic activities are fishing and farming. The Lake is the major source of water and mainly used for bathing, cooking, drinking and recreation. Because of high water contact, the residents have high risk of being infected with *S.mansoni* and carry highest infection intensity [15]. Annual mass drug administration (MDA) using praziquantel against *S. mansoni* infection mainly focus to schoolchildren and adult population is not included.

### Study design, inclusion and exclusion criteria

**Fig. 1:**
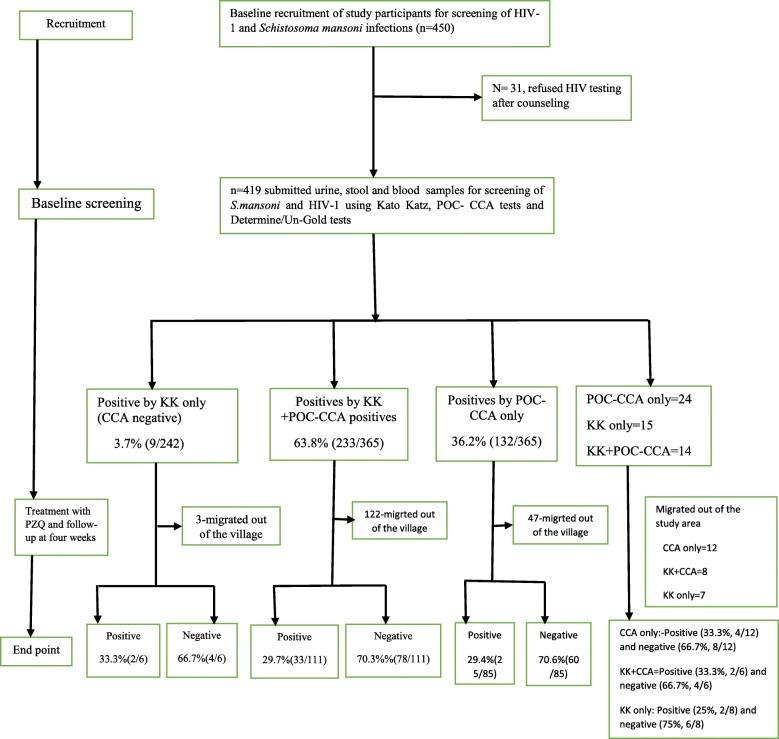
Study profile

This was a prospective longitudinal study, which was conducted in October–November 2016, was characterized with two cross-sectional surveys at baseline and 4 weeks after praziquantel treatment (Fig. 1). All adult individuals aged 15–55 years of age and lived in the study village for > 2 years were eligible for recruitment into the study. The length of stay in the risky areas for transmission of *S.mansoni* has been demonstrated to increase the chances for acquiring the infection and developing the related hepatosplenic morbidities [17]. The study excluded individuals who were on antiretroviral treatment (ART) due to the fact that excretion of *S.mansoni* eggs is a function of immune status as measured in term of CD4^+^ Th_2_ cells [9] and taking antiretroviral therapy have effects on the level of CD4^+^ Th_2_ cells [4]. Thus, including individuals who were on antiretroviral therapy would confound the results of Kato Katz technique among the HIV-1 infected group. The study also excluded individuals with CD4^+^ < 350cells/μL from the prospective survey. By the time this study was conducted, the country guideline for ART initiation required that all individuals diagnosed with HIV-1 infection and having CD4^+^ < 350cells/μL to be initiated treatment. Therefore, it was considered unethical to included HIV-1 infected individuals with CD4^+^ < 350cells/in a prospective study. Lastly, individuals with history of taking anti-schistosomiasis in the past 6-month were also excluded to remove the confounding effects of the treatment on the sensitivity of the diagnostic test especially parasitological technique.

### Sample size and sampling procedures

For calculation of the sample size, we used the formula for comparing two independent proportional to estimate sample size for comparing sensitivity/specificity of the POC-CCA test and that of the Kato Katz technique (referred as a Gold standard) [18]. At 95% confidence interval and a power of 80% to detect a difference of 10% from sensitivity of 85% (i.e. P_1_ = 85%, P_2_ = 75% and P_0_ = 77.5%) [18], the required sample size was 472 participants but only 419 participants were enrolled into the study. Although the sample size was below the estimated sample size, our power to detect the estimated difference remained > 90%.

Selection of the study participants has been describe in details in [15], briefly a random sampling procedure was used to select households and household members to participate in the study. Prior to study implementation, a list of households and household members was obtained to allow random sampling of eligible individuals from randomly selected households.

### Data collections

#### Questionnaires

A pre-tested questionnaire was used to collect demographic information of the study participants, past history of HIV testing, use of anti-schistosomiasis treatment and use of anti-retroviral therapy.

#### Examination of *Schistosoma mansoni* circulating cathodic (CCA) antigen and *Schistosoma haematobium* eggs

For screening of *S.mansoni* infection*,* a point-of-care Circulating-Cathodic-Antigen (CCA) (Rapid Medical Diagnostic- http://www.rapid-diagnostics.com/, Batch number: 33827) which uses urine sample was employed. A single urine sample was collected from each participating individuals [19] and the POC-CCA test was used to screen for the antigen. The POC-CCA test detects the presence of schistosome CCA released from worms excreted in the host’s urine [19]. The test was performed as per manufacturer instructions (Rapid Medical Diagnostic- http://www.rapid-diagnostics.com/) and “trace” results were considered as positive. Qualified laboratory technician were blinded for the Kato Katz parasitological results and HIV-1 sero-status of the study participants used the test to screen for *S.mansoni* infection.

For screening of *S.haematobium* infection, the same urine sample collected for POC-CCA screening was used. Urine sample was grossly examined for the presence of gross heamaturia and using Hemastix® for presence of micro-haematuria [20]. Then, the urine sample was filtered (pore aperture 20 μm; Sefar AG, Heiden, Switzerland), filter placed on a slide and examined under a microscope for presence of *S. haematobium* eggs [20].

#### Examination of human immunodeficiency Virus-1

Human Immunodeficiency Virus-1 (HIV-1) testing was done according to Tanzanian National HIV algorithm [21]. This involved the use of Determine® and UNGold qualitative tests [21]. Before, HIV testing, participants received counseling from qualified HIV counselors. Then, finger prick blood sample was collected from each consenting study participants and screening for HIV-1 infection using both test. Results were recorded based on the manufacturer instructions. The quantification of CD4^+^ cells was done using a FACS Calibur machine (Becton and Dickinson, BD Biosciences, San Jose, CA, USA), following standard manufacturer procedures.

#### Parasitological examination of *Schistosoma mansoni* eggs in stool samples

For diagnosis of *S. mansoni* infection, a single stool sample was collected from each study participant at baseline and 4 weeks after praziquantel treatment. On the screening day, each participating individual was provided with a container for stool sample collection. Stool samples were collected fresh, labeled with a specific study number and processed using Kato Katz thick smear technique using a template of 41.7 mg per thick smear [22]. From each collected stool sample, four Kato Katz thick smears were prepared. Three-laboratory technician supervised by a senior laboratory technician examined all Kato Katz thick smears at the National Institute for Medical Research, Mwanza, Tanzania. For quality assurance purposes, 10% of all positive and negative slides were re-examined by an independent laboratory technician.

#### Data analysis

A Microsoft Excel system was used for double data entry and data analysis was performed using Stata Version 15 (StataCorp, 2017, Stata statistical software: release 13. College Station, TX: StataCorp LP, Taxes, USA). Numbers and percentages summarized categorical variables. Comparison of proportions/categorical variables was done using chi-square (χ^2^)/fisher exact where appropriate. For continuous variables descriptive statistics were reported as means with standard deviation for normally distributed variable and medians with interquartile ranges (IQR) for variables that were not normally distributed. The arithmetic mean of *S. mansoni* egg counts for each participant was calculated from the counts of four Kato Katz thick smears and multiplied by 24 to obtain individual eggs per gram of faeces. *Schistosoma mansoni* egg counts were logarithmically transformed to check for normality but only non-transformed means are presented. Mean egg counts for *S. mansoni* between sex and age were compared using Student-t-test (two groups) or ANOVA (more than two groups). Intensity of infection was categorized according to WHO criteria as: 1–99 epg, 100–399 epg, ≥400epg defined as low, moderate and heavy intensities of infection respectively [23].

The sensitivity, specificity, positive and negative predictive values of the POC-CCA test was calculated using two gold standards, (1) egg-positive by Kato Katz technique (as reference standard for diagnosis of *S. mansoni* infection) and (2) combined gold standard (infection-positive by either Kato Katz technique or POC-CCA-positivity (assuming 100% specificity of the POC-CCA test) [14]. Specificity, i.e. the percentage of negative individuals correctly identified as such, was calculated as described in [24]. Similarly, sensitivity was calculated as percentage of positive individuals correctly identified as such [24]. In addition, positive predictive value (PPV = proportion of positive test results that are truly positive) and negative predictive value (NPV = proportion of negative test results that are truly negative) were calculated after calculating sensitivity and specificity of the diagnostic tests [24]. Statistical significance was assigned at *P* < 0.05 for all analyses.

#### Ethical consideration and confidentiality

Ethical approval was sought from the joint Ethical and Review Committee of Bugando Medical Centre and Catholic University of Health and Allied Sciences. The study received further permission from the district administrative and division authorities. Kiswahili translated informed consent forms were used to obtain participants consent to participate in the study and assent for those who were aged 15- < 18 years. Written informed consent was obtained from guardians/parents of all participants aged 15- < 18 years. For illiterate participants, a thumbprint was used to sign the consent/assent forms after a clear description of the study objectives was given to them. To maintain confidentiality, all the demographic and other clinical data of the study participants were kept in a closed cabinet and whenever the data were accessed no participants name were disclosed, only identification number of the participants were used to identify participants. All study participants infected with *S. mansoni* were treated with praziquantel (40 mg/kg) according to WHO guidelines [25].

## Results

### Demographic characteristics of study participants

A total of 419 study participants were enrolled in this study, of these 46.1% (*n* = 193/419) and 53.9% (*n* = 226/419) were female and male respectively. The median age of the study participants was 30 years (IQR: 22–42 years). Table 1 shows the age and sex distribution of the study participants from Igalagala village. All of the study participants were from Igalagala village located along the shoreline of the Lake Victoria. All of the study participants performed their economic activities within the village. The main economic activities of the study participants were farming, fishing and fish processing and small-scale business.

**Table 1:** Demographic characteristics (age and sex distribution) of the study participants

Sex	Age groups (in years)				
	15–20 years	21–30 years	31–40 years	41–50 years	51–55 years
Female	39 (46.9%)	63 (44.4%)	34 (41.9%)	26 (46.4%)	31 (54.4%)
Male	44 (53%)	79 (56.6%)	47 (58%)	30 (53.6%)	26 (45.6%)

### Prevalence of HIV-1 infection and determination of CD4^+^ levels

The overall prevalence of HIV-1 was 6.9% (*n* = 29/419, 95%CI: 4.8–9.8). The median CD4^+^ cell counts was 329cells/μL (Interquartile range: 299–526).

### Pre-treatment baseline prevalence and intensity of *Schistosoma mansoni* infection based on Kato Katz technique

Based on Kato Katz technique, the overall prevalence of *Schistosoma mansoni* infection was 57.8% (*n* = 242/419, 95%CI: 52.9–62.4), with female study participants having the highest prevalence than male participants (67.4% versus 49.6%, χ^2^ = 13.5186, *P* < 0.001). There was a relationship between age and *S.mansoni* infection, the youngest age group (14–20 years) had the highest prevalence compared to the oldest age group (51–55 years) which had lower prevalence compared to the youngest age groups (75.9% versus 36.8%, χ^2^ = 21.9706, *P* < 0.001). The overall arithmetic mean of egg intensity was 64.78 ± 161.08 (95%CI: 49.3–80.3). Female participants had higher arithmetic mean egg intensity compared to male participants (101.8 ± 209.6, 95%CI: 71.9–131.5 versus 33.21 ± 92.5, 95%CI: 21.1–45.3, F = 19.69, *P* < 0.001). Majority of the study participants had low infection intensity (70.7%), moderate (23.6%) and heavy infection intensity (5.8%). In general, more female participants than male participants had moderate to heavy infection intensity (χ^2^ = 12.9664, *P* < 0.002).

### Pre-treatment prevalence of *Schistosoma mansoni* infection based on point-of-care circulating Cathodic antigen tests

Based on the Point-of-Care Circulating Cathodic Antigen test, the overall prevalence of *S.mansoni* among adult participants was 87.4% (365/419, 95%CI: 83.5–90.0). Female individuals had the highest prevalence compare to male participants (91.2%, *n* = 176/193 versus 83.6%, *n* = 189/226, χ^2^ = 5.3043, *P* < 0.02). In relation to age, the youngest age group (15–20 years) had the highest prevalence of infection (χ^2^ = 10.6675, *P* < 0.03).

### Pre-treatment prevalence of *Schistosoma mansoni* infection based on point-of-care circulating Cathodic antigen tests and Kato Katz technique

Overall, a total of 233/365 (63.78%) study participants were detected to have *S. mansoni* infection by both Kato Katz technique and POC-CCA test. The POC-CCA test detected 96.3% (233/242) of all the study participants who had detectable *S. mansoni* eggs in their stool based on Kato Katz technique. The POC-CCA test detected an additional of 132 study participants infected with *S. mansoni* who were missed by Kato Katz technique.

### Co-infection of *Schistosoma mansoni* and HIV-1 infection

Based on Kato Katz technique, 6.2% (15/242) of the study participants were co-infected with HIV-1 and *S. mansoni* infection. Based on POC-CCA test, 6.6% (24/365) of the study participants were co-infected with HIV-1 and *S.mansoni* infection. Overall, 48.3% (14/29) of the HIV-1 seropositive participants were both KK/CCA positive for *S.mansoni* infection (meaning that 48.3% of study participants were HIV-1 positive and were identified by both KK and CCA to be infected with *S.mansoni*).

### Prevalence and intensity of *Schistosoma mansoni* 4 weeks after treatment based on Kato Katz technique and point-of-care circulating Cathodic antigen test

At 4 weeks follow-up after praziquantel treatment, a total of 202 study participants who were infected by *S. mansoni* based on either POC-CCA test or Kato Katz technique were available for re-examination. Of these participants, 111 participants were infected by *S.mansoni* based on POC-CCA test and Kato Katz technique at baseline, at 4 weeks after PZQ treatment, 70.3% (78/111) had no infection and only 29.7% (33/111) remained with *S.mansoni* infection based on both Kato Katz technique and POC-CCA test **(**Fig. 1**)**. For those who were identified to be infected by *S.mansoni* using POC-CCA test only at baseline, 85 were available for re-examination at 4 weeks after PZQ treatment, of these, 29.4%(25/85) remained POC-CCA positive for *S.mansoni* and 70.6% (60/85) had no *S.mansoni* infection. At baseline, only 9/242 (3.7%) of the study participants were identified by Kato Katz technique (had POC-CCA negative results) to be infected by *S.mansoni*. At 4 weeks after treatment, six (6) of them were available for re-examination, 4/6 (66.7%) had no infection and only 2/6(33.3%) remained with detectable eggs in their stool samples **(**Fig. 1**)**.

In relation to HIV-1 serostatus, of the 15 HIV-1 seropositive who were co-infected with *S.mansoni* infection based on Kato Katz technique, eight (8) of them were available for re-examination, 25% (2/8) remained with *S.mansoni* eggs in their stool samples at 4 weeks after treatment **(**Fig. 1**)**. Based on POC-CCA test, of the 24 HIV-1 seropositive who had POC-CCA positive test at baseline, 12 were available for re-examination, of these 33.3% (4/12) remained with CCA positive results at 4 weeks of follow-up **(**Fig. 1**)**. There was no difference in age and sex differences between those who were present and those who were absent at 4 weeks of follow-up (χ^2^ = 5.7496, *P* = 0.23). In addition, there was a mean difference in infection intensity between those who were present and those who were absent at 4 weeks of follow-up (*t* = 2.3329, *P* < 0.02), with those who were absent had a higher infection intensity.

### Diagnostic characteristics of the point-of-care circulating cathodic antigen test before treatment

Table 2 shows pre-treatment sensitivity and specificity of POC-CCA test using Kato Katz technique as a “gold standard”. At baseline, the sensitivity and specificity of POC-CCA test were 96.3% (95%CI: 93.1–98.3) and 25.4% (95%CI:19.2–32.5) for the general population. The negative and positive predictive values for the general population are shown in Table 2. The sensitivity and specificity of the POC-CCA test using the combined gold standard (infection-positivity by either egg-or-POC-CCA positivity, assuming 100% specificity of the POC-CCA test) are shown in Table 2. The overall sensitivity slightly improved from 96.3%(95%CI: 93.1–98.3) to 97.6% (95%CI: 95.5–98.9) for the general population.

**Table 2:** Diagnostic characteristics of the Point-of-care Circulating Cathodic Antigen test using Kato Katz technique as gold standard and combined gold standard in the general population and HIV-1 seropositive group before and 4 weeks after treatment

Diagnostic test	Sensitivity	Specificity	Positive Predictive Value	Negative Predictive Value
Pre-treatment				
General population (i) Using Kato Katz technique as gold standard				
POC-CCA	96.3% (95%CI:93.1–98.3)	25.4% (95%CI: 19.2–32.5)	63.8% (95%CI: 58.7–68.8)	83.3% (95%CI: 70.7–92.1)
(ii) Using combined gold standard^a^				
POC-CCA	97.6% (95%CI:95.5–98.9)	100 (95%CI:92.1–100)	100 (95%CI:99–100)	83.3% (95%CI:70.7–92.1)
In the HIV-1 seropositive group (i) Using Kato Katz technique as a gold standard				
POC-CCA	93.3% (95%CI:68.1–99.8)	28.6% (95%CI:8.4–58.1)	58.8% (95%CI:36.6–77.9)	80% (95%CI:28.4–99.5)
(ii) Using combined gold standard^a^				
POC-CCA	96% (95%CI:79.6–99.9)	100% (95%CI:39.8–100)	100% (95%CI:85.8–100)	80% (95%CI:28.4–99.5)
Four weeks after praziquantel treatment				
In general population (i) Using Kato Katz technique as gold standard				
POC-CCA	47.8% (95%CI: 26.8–69.4)	74.7% (95%CI: 67.9–80.8)	19% (95%CI: 9.9–31.4)	92.1% (95%CI: 86.5–95.8)
(ii) Using combined gold standard^a^				
POC-CCA	84.4% (95%CI:74.4–91.7)	100% (95%CI:97.4–100)	100% (95%CI:94.5–100)	92.1% (95%CI:86.5–95.8)
In the HIV-1 seropositive group				
(i) Using Kato Katz technique as gold standard				
POC-CCA	100% (95%CI:2.5–100)	63.6% (95%CI:30.8–89.1)	20% (95%CI:0.5–71.6)	100% (95%CI:59–100)

POC-CCA-Point-of-Care Circulating Cathodic Antigen

^a^Infection-positive by either egg-or-POC-CCA-positivity (assuming 100% specificity of the POC-CCA result)

In relation to HIV-1 serostatus, the results obtained using Kato Katz technique as a gold standard and combined gold standard are shown in Table 2. Using the Kato Katz technique as a gold standard, the sensitivity of POC-CCA test was 93.3% (95%CI: 68.1–98.8) and specificity was 28.6% (95%CI: 8.4–58.1), Table 2. Then, Using the combined gold standard (infection-positivity by either egg-or-POC-CCA positivity, assuming 100% specificity of the POC-CCA test), the sensitivity of the POC-CCA test improved to 96.0% (95%CI: 79.6–99.9).

### Diagnostic characteristics of the point-of-care circulating cathodic antigen test after treatment

The results obtained using Kato Katz technique as a gold standard and combined gold standard (infection-positivity by either egg-or-POC-CCA positivity, assuming 100% specificity of the POC-CCA test) 4 weeks after treatment are shown in Table 2**.** After 4 weeks of treatment, using Kato Katz technique as a gold standard, the overall the sensitivity and specificity of POC-CCA test were 47.8% (95%CI: 26.8–69.4) and 74.7% (95%CI: 67.9–80.8) respectively. Using the combined gold standard, at 4 weeks after treatment, the overall sensitivity of the POC-CCA test was 84.4% (95%CI: 74.4–91.7) (Table 2).

### Diagnostic characteristics of the Kato Katz technique compared to combined gold standard pre-and-post treatment

At pre-treatment, the sensitivity of the Kato Katz technique compared to combined gold standard was 64.7%(95%CI: 59.6–69.5), Table 3. Four weeks after treatment, a very low sensitivity of Kato Katz technique was recorded at 35.6% (95%CI: 24.7–47.7).

**Table 3:** Diagnostic characteristics of the Kato Katz technique using a combined gold standard in the general population and HIV-1 seropositive group before and 4 weeks after treatment

Diagnostic test	Sensitivity	Specificity	Positive predictive value	Negative predictive value
General population				
Pre-treatment combined gold standard^a^				
Kato Katz	64.7% (95%CI:59.6–69.5)	100% (95%CI:92.1–100)	100% (95%CI:98.5–100)	25.4% (19.2–32.5)
Post-treatment combined gold standard^a^				
Kato Katz	35.6% (95%CI:24.7–47.7)	100% (95%CI:97.4–100)	100 (95%CI:86.8–100)	74.7% (95%CI:67.9–80.8)
In the HIV-1 seropositive group				
Pre-treatment combined gold standard^a^				
Kato Katz	60% (95%CI:38.7–78.9)	100% (95%CI:39.8–100)	100% (95%CI:78.2–100)	28.6% (95%CI:8.4–58.1)
Post-treatment combined gold standard^a^				
Kato Katz	33.3% (95%CI:43–77.7)	100% (95%CI:59–100)	100% (95%CI:15.8–100)	63.6% (95%CI:30.8–89.1)

^a^Infection-positive by either egg-or-POC-CCA-positivity (assuming 100% specificity of the Kato Katz result)

## Discussion

The main findings from this study indicate that, pre-and-post treatment sensitivity and specificity of the point-of-care circulating Cathodic Antigen test using combine gold standard were higher than that of Kato Katz technique, both in the general population and the HIV-1 seropositive group. We observed declined in sensitivity of POC-CCA test 4 weeks after treatment, when KK technique was used as a gold standard, but this changed to a higher sensitivity when a combined gold standard was used. We observed high praziquantel cure rates both in HIV-1 seropositive and seronegative groups infected with *S.mansoni* evaluated at 4 weeks after treatment.

At pre-treatment assessment, our data indicated that POC-CCA test achieved a sensitivity of 96.3% but had low specificity in detecting *S.mansoni* infection using Kato Katz technique as a gold standard in the general population. Similar findings were noted by previous similar studies conducted elsewhere in African setting using Kato Katz technique as a gold standard [26, 27]. Conversely, at pre-treatment, the POC-CCA test achieved high sensitivity (93.3%) but low specificity in detecting *S.mansoni* infection in the HIV-1 seropositive group. A high pre-treatment intensity of *S.mansoni* infection appears to influence the high sensitivity of POC-CCA test [28]. Significant positive correlations between positivity of POC-CCA test and *S. mansoni* eggs counts have been noted in previous study in Brazil and Kenya [28, 29]. This partly could explain a high pre-treatment sensitivity of the POC-CCA test in the two groups, which is reported to decrease in individuals with light infections (< 100 eggs/g of feaces) [26, 28]. The low POC-CCA test specificities in part are explained by the low sensitivities of the Kato Katz technique [26]. Our pre-treatment findings on the sensitivity and specificity of POC-CCA test are comparable to previous findings published among primary school children in endemic areas of Uganda (94.8%) [26] but higher than 86.9% and 56.3% observed in Cote d’Ivoire [27]. The variation in prevalence and intensity of *S.mansoni* infection between geographical settings seems to influence the sensitivity of the POC-CCA test. In region with low prevalence such as in Cote d’Ivoire, the sensitivity of POC-CCA test was lower (56.3%) despite that three POC-CCAs were performed to increase sensitivity [27]. In our present study, 32.5% and 35% of the study participants identified by POC-CCA test having *S.mansoni* infection in the general population and in the HIV-1 seropositive group were missed by Kato Katz technique. This observation indicate that, if selective treatment is done based on the KK test, these individuals would be erroneously indicated not infected. Previous studies have demonstrated reduce efficiency of *S.mansoni* eggs excretion in HIV-1 individuals co-infected with *S.mansoni* infection [1, 9, 15, 30]. Thus, the use of a more sensitive diagnostic test/technique than Kato Katz technique is justifiable in adult population likely to be co-infected with HIV-1 and *S.mansoni* and likely to have light to moderate intensity of infection [26].

At 4 weeks post-treatment using Kato Katz technique as a gold standard, the sensitivity of POC-CCA test decreased to below 50% but its specificity improved to almost to 75% in the general population. A similar findings have been reported in Uganda, in which at 1 and 4 weeks post-praziquantel treatment, the sensitivity of the POC-CCA test decreased from 91.7% to 66.7% and 73% [26]. A plausible explanation for this observation is the increase in POC-CCA false positives due to increase in specificity [26, 29, 31]. Alternatively, the decline in intensity of infection at post-praziquantel treatment affects the performance of POC-CCA tests [28]. A noted pre-treatment correlation between *S.mansoni* egg loads and the strength of positivity of POC-CCA test has been reported [26, 29, 32]. An interesting questions which needs follow-up is the relationship between parasites antigen levels at pre-and-post praziquantel treatment and reduced eggs intensity following praziquantel treatment. The fact that Circulating Cathodic Antigen are released by mature adult worms which are also responsible for producing eggs [19], it will be interesting to understanding this relationship.

In the HIV-1 seropositive group, the POC-CCA test attained a sensitivity of 100% using Kato Katz technique as a gold standard. Is worthwhile mentioning that at 4 weeks post-treatment evaluation, our analysis suffered from low number of HIV-1/*S.mansoni* co-infected individuals to give a meaningful statistical analysis on the sensitivity and specificity of the POC-CCA test. This is a limitation for this study. Evidence from our data indicate that for the HIV-1/*S.mansoni* co-infected individuals who returned after 4 weeks for re-examination, at least 60% were Kato-Katz negative/POC-CCA positive. Similar findings have been reported in rural Zimbabwe, where a low CCA clearance rates was observed in HIV-1/*S.mansoni* co-infected individuals [30]. In term of public health, this findings adds weight on the need to included highly sensitive diagnostic test especially when screening and evaluating MDA programs which involve adult individuals in areas which are co-endemic to HIV-1/*S.mansoni* infection.

Understanding the limitations of Kato Katz technique as gold standard diagnostic test [33], we developed an assumption that the POC-CCA test is 100% specific and constructed an artificial gold standard based on either Kato Katz technique egg positive or POC-CCA test seropositivity [14]. Using the combined gold standard resulted in improved in the sensitivity of POC-CCA test compared to what was observed when using Kato Katz technique as a gold standard, both at pre-treatment (96.3% versus 97.6%) and 4 weeks after treatment (47.8% versus 84.4%) in the general population. Conversely, for the HIV-1 seropositive group, the use of combined gold standard resulted in increased in sensitivity of POC-CCA test compared to what was recorded when using Kato Katz technique before treatment (93.3% versus 96%). However, using a combined gold standard, the sensitivity of Kato Katz technique before and 4 weeks after treatment, both in the general population and in the HIV-1 seropositive group remained lower than that of POC-CCA test. These findings indicates the needs to supplement Kato Katz technique in either epidemiological surveys or when assessing the efficacy of praziquantel drug especially in adult individuals likely to have light infection intensity or co-infected with HIV-1/*S.mansoni*. The use of more than one diagnostic tests to screen parasitic diseases is highly recommended in order to improve the sensitivity of field based diagnostic techniques. This increases the chances of detecting individuals with different levels of infection intensity in endemic areas and allows precise measure of the burden of disease when planning for community based intervention measures [34, 35].

Efficacy of praziquantel drugs as measured by parasitological cure rates between the HIV-1 seropositive groups co-infected with *S.mansoni* and HIV-1 seronegative group infected only with *S.mansoni* parasite did not vary significantly. Similar findings were reported in northwestern Tanzania, in which parasitological cure rates of 62.6% and 48.3% were reported among HIV-1 seronegative group infected with *S.mansoni* and HIV-1-*S.mansoni* co-infected group [36]. At 12 weeks assessment of praziquantel treatment, Kallestrup et al., [13] in Zimbabwe recorded parasitological cure rates of 86% and 85% in HIV-1 seropositive group co-infected with *S.mansoni* and those only infected with *S.mansoni* [13]. However, at 4 weeks of praziquantel treatment evaluation, Karanja et al., in [12] recorded lower cure rates of 53% and 59% among HIV-1 seronegative group infected with *S.mansoni* and those who were co-infected with HIV-1 and *S.mansoni* [22]. The variation in pre-treatment intensity of *S.mansoni*, intensity of transmission and low sensitivity to praziquantel in some of the strains of *S.mansoni* parasite can partly explains the observed differences in cure rates between the HIV-1 seronegative groups and groups co-infected with HIV-1 and *S.mansoni* [37].

The observation of Kato Katz-negative/POC-CCA positive results at 4 weeks post-praziquantel treatment may reflects existing of adult or juvenile stages not affected by treatment and newly acquired infection in high transmission setting, like that of the present study area. In general, the presence of Kato Katz negative/POC-CCA positive reflects continued infection in treated individuals (having worms which continue to release CCA). With majority of Kato Katz negative individuals remaining harboring light intensity of infections as shown by POC-CCA test in the present study and those of other authors [26, 29], these findings clearly suggest the need for repeated rounds of treatment for persistent infection per year contrasting the current recommendation of once per year [23]. This means more costs to the MDA programs. To reduce costs, the currently used Kato Katz technique to evaluate MDA program need to be supplemented with a more sensitive assay such as POC-CCA test.

The present study was not conducted without limitation, the low number of HIV-1/*S.mansoni* co-infected individuals affected our analysis on the evaluation of sensitivity and specificity of POC-CCA test and Kato Katz technique at 4 weeks after treatment. Moreover, the use of a single stool sample to examine for *S.mansoni* infection owing the day to day variability of *S.mansoni* eggs output may have affected the performance of the Kato Katz technique.

## Conclusion

Our findings indicate that the POC-CCA test had higher sensitivity at pre-and-post praziquantel treatment than Kato Katz technique using a combined gold standard. The sensitivity of POC-CCA test remained higher than Kato-Katz technique even in the presence of HIV-1 co-infection before and after praziquantel treatment. However, the POC-CCA test had low specificity at pre-treatment which increased at 4 weeks post-praziquantel treatment. Although POC-CCA test have shown promising results for its use in post-treatment evaluation of MDA program, further studies are needed to elucidate the full potential of the test in evaluating the impact of MDA programs in population living in areas with different transmission intensity.

## Abbreviations

CD4: Cluster of differentiation number four
IQR: Interquartile range
KK: Kato Katz technique
MDA: Mass Drug Administration
NPV: Negative predictive value
POC-CCA: Point-of-care Circulating Cathodic Antigen
PPV: Positive predictive values
WHO: World Health Organization
